# Supplementary material for: The causal role of circulating inflammatory markers in osteoporosis: a bidirectional Mendelian randomized study
Source: Front Immunol. 2024 Jul 18;15:1412298. doi: 10.3389/fimmu.2024.1412298 (PMC11291241; doi:10.3389/fimmu.2024.1412298)
Supplement: Supplementary file 1 [file DataSheet_1.zip › supplementary material/OSTEOPOROSIS-CIM/GCST90274810.tsv.gz_forest.pdf]

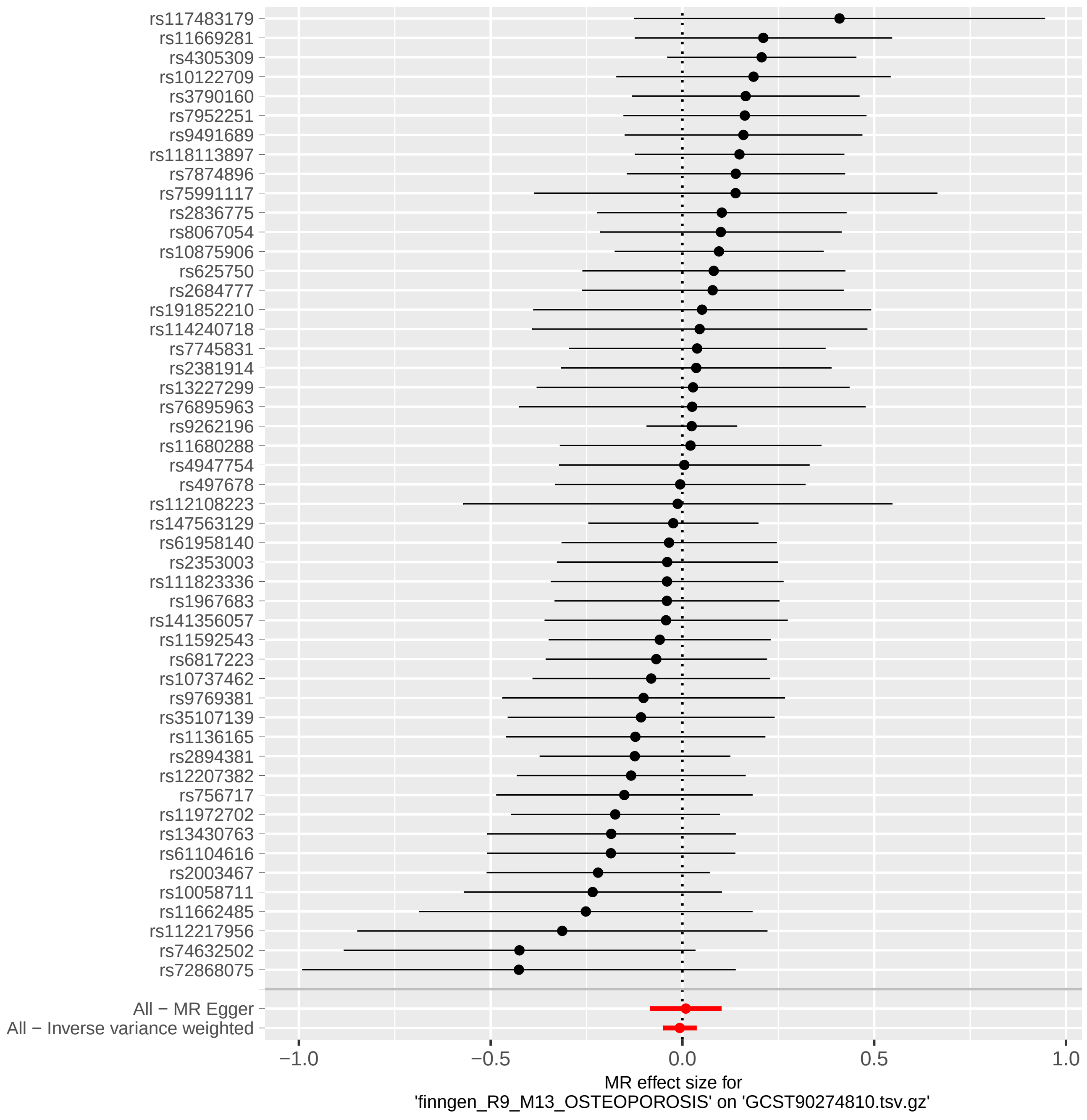
rs117483179
rs11669281
rs4305309
rs10122709
rs3790160
rs7952251
rs9491689
rs118113897
rs7874896
rs75991117
rs2836775
rs8067054
rs10875906
rs625750
rs2684777
rs191852210
rs114240718
rs7745831
rs2381914
rs13227299
rs76895963
rs9262196
rs11680288
rs4947754
rs497678
rs112108223
rs147563129
rs61958140
rs2353003
rs111823336
rs1967683
rs141356057
rs11592543
rs6817223
rs10737462
rs9769381
rs35107139
rs1136165
rs2894381
rs12207382
rs756717
rs11972702
rs13430763
rs61104616
rs2003467
rs10058711
rs11662485
rs112217956
rs74632502
rs72868075
All − MR Egger
All − Inverse variance weighted
−1.0
−0.5
0.0
0.5
1.0
MR effect size for
'finngen_R9_M13_OSTEOPOROSIS' on 'GCST90274810.tsv.gz'
